# Microbiota-host interplay at the gut epithelial level, health and nutrition

**DOI:** 10.1186/s40104-016-0123-7

**Published:** 2016-11-08

**Authors:** Jean-Paul Lallès

**Affiliations:** 1 Division of Human Nutrition Division, INRA Clermont-Ferrand, France; 2 Human Nutrition Research Center – West, Nantes, France; 3 Present Address: INRA – SDAR, Domaine de la Motte, B.P. 35327, F-35653 Le Rheu Cedex, France

**Keywords:** Diet, Gut, Inducible heat shock protein, Inflammation, Intestinal alkaline phosphatase, Microbiota

## Abstract

Growing evidence suggests the implication of the gut microbiota in various facets of health and disease. In this review, the focus is put on microbiota-host molecular cross-talk at the gut epithelial level with special emphasis on two defense systems: intestinal alkaline phosphatase (IAP) and inducible heat shock proteins (iHSPs). Both IAP and iHSPs are induced by various microbial structural components (e.g. lipopolysaccharide, flagellin, CpG DNA motifs), metabolites (e.g. n-butyrate) or secreted signal molecules (e.g., toxins, various peptides, polyphosphate). IAP is produced in the small intestine and secreted into the lumen and in the interior milieu. It detoxifies microbial components by dephosphorylation and, therefore, down-regulates microbe-induced inflammation mainly by inhibiting NF-κB pro-inflammatory pathway in enterocytes. IAP gene expression and enzyme activity are influenced by the gut microbiota. Conversely, IAP controls gut microbiota composition both directly, and indirectly though the detoxification of pro-inflammatory free luminal adenosine triphosphate and inflammation inhibition. Inducible HSPs are expressed by gut epithelial cells in proportion to the microbial load along the gastro-intestinal tract. They are also induced by various microbial components, metabolites and secreted molecules. Whether iHSPs contribute to shape the gut microbiota is presently unknown. Both systems display strong anti-inflammatory and anti-oxidant properties that are protective to the gut and the host. Importantly, epithelial gene expressions and protein concentrations of IAP and iHSPs can be stimulated by probiotics, prebiotics and a large variety of dietary components, including macronutrients (protein and amino acids, especially L-glutamine, fat, fiber), and specific minerals (e.g. calcium) and vitamins (e.g. vitamins K1 and K2). Some food components (e.g. lectins, soybean proteins, various polyphenols) may inhibit or disturb these systems. The general cellular and molecular mechanisms involved in the microbiota-host epithelial crosstalk and subsequent gut protection through IAP and iHSPs are reviewed along with their nutritional modulation. Special emphasis is also given to the pig, an economically important species and valuable biomedical model.

## Background

The gastrointestinal tract (GIT) is, like the skin or the lung, a major interface organ between the environment and interior milieu. It is the site with the highest load of microorganisms (also referred to as “the microbiota”). This is especially true in the large intestine due to substantial amounts of undigested dietary and endogenous (e.g. mucus, enzymes) components amenable to microbial fermentation. Gut epithelial cells are thus the first cells to be exposed to nutrients and the microbiota, with complementary functions between the small intestine aiming at digestion and nutrient absorption and the large intestine specialized in the fermentation of undigested materials. The gut epithelium is also the first line of GIT (and body) defense and protection. Its action is complementary to that of the associated mucosal immune system whose development and maintenance are induced by the microbiota [1]. Thus gut epithelial cells - enterocytes and colonocytes - are polarized key players influenced by both the environment (e.g. food, pathogens, toxicants) and body metabolism and functions. The gut epithelium has developed over time various mechanisms for sensing not only nutrients but also microbial structural components (e.g. lipopolysaccharide, LPS; peptidoglycan, flagellin, CpG DNA motifs), metabolites (e.g. short chain fatty acids, SCFA) or secreted molecules (e.g. toxins, polyphosphate chains, other compounds still unknown). These sensors include for example Toll-like receptors (TLRs) [2, 3] and receptors to SCFA. All these mechanisms make the molecular basis of the crosstalk between the host and the gut microbiota at the epithelial level.

Numerous experimental and clinical data have shown that defects in gut barrier function may lead to chronic inflammatory diseases and sometimes cancers [4–7]. These diseases affect not only the GIT but also other organs (e.g. liver, brain) and include diverse metabolic disturbances (ranging from glucose intolerance and insulin resistance, type-2 diabetes to metabolic syndrome and obesity), known risk factors for cardiovascular disorders. Importantly, more recent investigations has highlighted that many of these diseases may be modulated by the gut microbiota [8], though cause-and-effects relationships are often poorly understood. For instance, chronic metabolic diseases and obesity may be related to body entry of enteric microbial components (e.g. LPS) thus triggering chronic low-grade, “metabolic” inflammation [9, 10]. This in turn favors diet energy extraction, fat synthesis and adipose tissue development, and shifts energy metabolism towards fat deposition and adipose tissue inflammation, thus leading to metabolic syndrome and obesity. The diet is a major lever of gut microbiota modulation and is now regarded as a serious approach for maintaining high microbiota diversity (or gene richness) and preserving health as well as correcting dysbiosis often observed in many chronic diseases [11]. This is of utmost importance in the context of drastic reduction of food diversity over the last decades [12].

The present review focuses on two specialized defense and protection systems at the epithelial level, namely intestinal alkaline phosphatase (IAP) and inducible heat shock proteins (iHSPs). Both of them are modulated by the microbiota and the diet and confer gut epithelial (and body) protection due to their potent anti-inflammatory and anti-oxidant capacities. Data available in the pig are also reviewed given the economic importance of this species and its high potential as a biomedical model for studies on development, microbiology, physiology, neurobiology and nutrition [13–16]. In particular, the weaning period is critical to pig rearing due to high stress, GIT pathophysiology, growth check and increased risk of enteric diseases [17, 18]. Fortunately, selected dietary approaches may help circumvent these disorders [19]. Therefore, dietary components improving gut health through stimulating IAP and inducible HSP proteins are briefly reviewed here too.

### Intestinal alkaline phosphatase and the gut microbiota

Intestinal alkaline phosphatase (IAP), the specific intestinal isoform of ubiquitous AP gene products, displays an array of physiological properties that include: enterocyte apical surface pH maintenance through the control of bicarbonate secretion, absorption of nutrients and minerals (e.g. fatty acids, calcium), detoxification (by dephosphorylation) of pro-inflammatory microbial components (e.g. LPS, flagellin, CpG DNA motifs, uridine diphsophate (UDP)) and, ultimately control of gut (and systemic) inflammation [20, 21]. IAP is an enzyme dynamically produced by the enterocyte in the small intestine and secreted both luminally and basolaterally. Part of lumen IAP escapes digestion in the bowel, remains active along the large intestine and can still be detected in small amounts in the feces.

Previous data suggested IAP to participate indirectly to the control of intestinal barrier function [21], but a direct involvement was demonstrated in mice recently [22]. More precisely, IAP stimulates gene expression of key tight junctions (Zonula occludens ZO-1 and ZO-2; occludin) and their correct cellular localization.

Many recent data now converge to indicate that IAP not only detoxifies microbial components but also contributes to shape the gut microbiota and to prevent microbial enteric translocation into the body [14]. Free exogenous (e.g. from bovine intestine) IAP per se does not seem to influence bacterial growth but enterocyte-bound IAP could delay that of *Escherichia coli* *in vitro* (with no effects on other bacteria such as *Clostridium difficile, S. typhimurium or Enterococcus faecalis*) [23–25]. Mice deleted for *Iap* gene (called *Akp3* in this species) were reported to display fecal microbiota that were different from those of wild-type mice: marked decrease in the overall load of both aerobic and anaerobic bacteria, drastic reduction in *E. coli* population and, conversely, increases in *Clostridiales*, *Lactobacilli* and *Enterococci* [24]. The precise mechanisms for these IAP-dependent changes in gut microbiota composition are not fully understood yet but they may involve alterations in epithelial surface pH and reduced gut inflammatory tone [26, 27]. Another pathway of microbial control involving IAP was recently reported [28, 29]. Free luminal adenosine triphosphate (ATP), a strong pro-inflammatory danger signal, dose-dependently inhibited microbial growth, targeting more specifically Gram-positive (but not Gram-negative) bacteria [29]. IAP was able to dephosphorylate and detoxify ATP, thus ultimately releasing free adenosine which is a strong anti-inflammatory molecule. Importantly, ATP was shown to drive cell differentiation of Th17 T lymphocytes that produce IL-17 and IL-22 cytokines. The former is known to favor neutrophil tissue infiltration while both cytokines stimulate antibacterial peptide production. IAP was already shown to inhibit gut tissue infiltration of neutrophils in zebra fish [23], thus strengthening the anti-inflammatory capabilities of IAP.

Regarding bacterial translocation, earlier investigations reported an inhibitory effect of IAP [30]. However, later work suggested a rather indirect influence though IAP-driven down-regulation of inflammation and subsequent reinforcement of gut barrier function [31, 32].

*Collectively these data indicate that IAP directly and indirectly controls gut microbiota load and balance and that this directly connects to gut inflammatory tone*.

### Inducible heat shock proteins and the gut microbiota

Beside the general roles of HSPs as intracellular protein chaperones, those induced specifically in gut epithelial cells, namely HSP25 (or HSP27, depending on the host species) and HSP70 are involved in many vital functions (e.g. cell proliferation and apoptosis, immune responses) and the control of inflammation and oxidation [33, 34]. Importantly, iHSPs regulate gut barrier function, by specifically controlling the expression of key tight junction proteins (e.g. occludin) and by down-regulating adverse effects of oxidative and inflammatory stress on cells [33].

In rodents, epithelial iHSPs are expressed at low and high levels in the small and large intestines, respectively [34]. This actually reflects the loads of microbes present along these compartments and that are a major factor of iHSP induction. Indeed, intestinal and colonic epithelial cells per se are equally responsive to iHSP-inducing stimuli and the gut proximal-distal iHSP gradient disappears in germfree animals [35, 36].

The microbiota-host epithelial crosstalk is first brought about by specific microbial compounds, including structural components (e.g. LPS, lipoteichoic acid, flagellin), metabolites (especially n-butyrate but also propionate), toxins (e.g. toxin A from *Clostridium difficile*, enterotoxin B superantigen from *Staphylococcus aureus*) and other soluble substances (e.g. various peptides like fMLP) [34]. All these substances are recognized by specific receptors (e.g. TLRs) or are internalized in gut epithelial cells by specific transporters (e.g. the peptide transporter PepT1), and intracellular signaling pathways involve various kinases (especially p38 MAPK) [34]. Many HSP inducers are active at very low concentrations (ng order) and responses are often fast (within a few hours). Therefore, the physiological epithelial iHSP tone is under direct influence of the gut microbiota composition and metabolic activities. Their stimuli are, in turn, essential for permanently triggering optimal levels of epithelial defense given the fact that iHSPs confer protection to gut epithelial cells exposed to oxidative stress and inflammation [34].

Anaerobic bacteria (e.g. *Bacteroides fragilis*) were reported to have important roles in HSP induction [37, 38]. A variety of Gram-negative bacteria (e.g. *E. coli*) and Gram-positive bacteria (*Bifidobacterium breve, Lactobacillus paracasei, L. plantarum, L. Johnsonii*) have been shown to be strong inducers of gut epithelial iHSPs *in vitro* and sometimes *in vivo*, though others (e.g. *Enterobacter aerogenes* and *Proteus mirabilis* for Gram-negative species; *Enterococcus faecalis* for Gram-positive species) had no effects on iHSPs. In the same line, many probiotics, especially of *Lactobacilli* and *Bifidobacteria* strains, but not all probiotics (e.g. *E. coli* Nissle 1917) were demonstrated to induce gut epithelial HSPs and different cell sensors (e.g. TLRs or other molecules) and signaling pathways (often p 38 MAPK) have been disclosed (Table 1) (see also Table 2 and Table of ref. [34]). Finally, some (e.g. metronidazole), but not all antibiotics (or mixtures) may decrease iHSP levels and increase gut susceptibility to microbial toxins (e.g. *C. difficile* toxin A).

**Table 1:** Molecular sensors, microbial component and intracellular signalling pathways involved in the induction of HSPs by intestinal epithelial cells (adapted from ref. [34])

Molecular sensor/receptor on intestinal epithelial cell	Microbial component recognized	Signalling pathway involved
TLR-2	Lipoteichoic acid	?
TLR-4	Lipopolysaccharide	MAPK p38, ERK1/2
TLR-5	Flagellin	MAPK p38
GPR-41 & GPR-43 (putatively)	Butyrate, propionate	?
PepT1	fMLP peptide	MAPK p38
OCTN-2	ERGMT peptide	MAPK p38
Integrin-β	Polyphosphate chains	MAPK p38

*Collectively these data suggest that iHSP induction at the gut level might be one important mechanism of gut epithelial protection by commensal bacteria and probiotics and that any alterations in this protection may be detrimental to the host.*

### Dietary modulation of gut defense and protection systems

We have reviewed that many dietary compounds can modulate both IAP and iHSP gene expressions and protein concentrations or activities [20, 21, 34].

#### Intestinal alkaline phosphatase

Food intake per se is a stimulator of IAP while starvation has opposing effects [30]. Dietary added calcium stimulates IAP in rat intestine [39]. Calcium is known to be protective in colonic inflammation models but the implication of IAP was not explored. Free phosphorus had inhibitory effects on IAP while bound phosphate (e.g. to starch in some potato varieties) is dose-dependently stimulatory. Therefore, calcium-to-phosphorus ratio and their chemical forms in the diet are critical to IAP activity. Besides, vitamins K1 (philloquinone) and K2 (menaquinone-4) could also stimulate IAP in rodents.

Fat intake stimulates IAP in rodents and this has been interpreted as an adaptive response to fat-dependent increases in intestinal LPS uptake and translocation (via the chylomicron pathway) into the interior milieu [40]. The degree of saturation and length of fatty acids are also important to consider [20, 21]. Saturated and medium-chain fatty acids appear as stronger inducers of IAP compared to poly-unsaturated fatty acids (PUFA). Saturated fats are known for shifting the gut microbiota towards more Gram-negative bacteria and, therefore, more pro-inflammatory microbial components and more inflammation [41]. Importantly, intestinal tissue concentration of (n-3) PUFA was recently demonstrated to determine the level of gene expression and enzyme activity of IAP which, in turn modified the gut microbiota composition and enhances barrier function [42]. In particular, the proteobacteria phylum (e.g. *E. coli* and other LPS-producing species) was reduced while anti-inflammatory bacteria (e.g. *Bifidobacteria*, *Lactobacilli*; *Akkermansia muciniphila*) were enhanced in (n-6) PUFA-fed, genetically engineered (Fat-1) mice that are able to convert dietary (n-6) PUFA into (n-3) PUFA. This contributes to explain, especially at the gut level the anti-inflammatory properties of (n-3) PUFA.

#### Inducible gut epithelial HSPs

Many dietary components are modulators of gut epithelial iHSPs [43]. This includes notably various amino acids and proteins, fiber, zinc, n-butyrate and many probiotics. The stronger inducer of iHSPs is without contest L-glutamine whose action is fast and of high magnitude. Its mode of action involves polyamines that increase the binding between transcription factor HSF-1 and heat-shock element on *Hsp* genes. Putrescine and spermidine, and their precursor ornithine stimulate the induction of both HSP25 and HSP70 in various gut epithelial cell lines *in vitro*. Spermine seems to induce HSP25 only. Molecular mechanisms of L-glutamine action involve the up-regulation of *Hsf1* gene expression and promoter activation resulting in iHSP production and subsequent down-regulation of the pro-inflammatory NF-κB pathway (by inhibiting protein p65 nuclear translocation and cell apoptosis). Other iHSP-stimulatory L-amino acids, though less effective than glutamine include glutamate, arginine, threonine and metabolic intermediates like citrulline [34]. Regarding dietary proteins, plant lectins (from kidney bean or wheat germ) inhibit iHSP expression while wheat gluten (involved e.g. in celiac disease) disturbs iHSP cellular localization *in vitro*, thus increasing cell sensitivity to oxidation and inflammation.

Mineral and organic forms of zinc as well as SCFA like butyrate (n- and iso- forms) and propionate are strong inducers of gut epithelial iHSPs *in vitro*. Pectin, a soluble and fermentable fiber (but not cellulose) stimulates both iHSPs in the ileum and the colon of rats. Conversely, pro-inflammatory, high sulfated saccharides like dextran sulfate sodium and carrageenans are known to disturb iHSP phosphorylation and functionalities, thus favoring gut inflammation. Therefore, the type of dietary fiber is important to consider when iHSP stimulation is needed. Surprisingly, various polyphenols were often shown to be potent inhibitors of gut iHSPs (e.g. quercetin), though they display anti-oxidant properties [34]. Finally, dietary mycotoxins with high oxidant capacity (e.g. zearalenone, fumonisins) induce iHSPs but this response is usually insufficient to counteract mycotoxin toxicity.

Many probiotics, especially *Lactobacillus* and *Bifidobacteria* strains are inducers of gut epithelial iHSPs and contribute to gut protection (see Tables 3 and 5 in ref. [34]). These probiotics can induce either or both (HSP25 and HSP70) iHSPs, depending on the strain. Inhibition of pro-inflammatory cytokine (e.g. IL-8) secretion and of some pathogens (e.g. *S. typhimurium*) has been documented too. The probiotic-dependent protection are mediated by various microbial triggers: cell wall components (lipoteichoic acids, LPS, flagellins), metabolites (butyrate, propionate) or secreted molecules (e.g. peptides; polyphosphate) (Table 1). A number of epithelial cell membrane sensors have been identified (TLRs, peptide transporters, etc.) while others remain to be discovered. Intracellular signaling often involves kinases, and especially p38 MAPK. Interestingly, Japanese groups have selected Lactobacillus (*L. paracasei* and *L. brevi*) probiotic strains that produce high amounts of long-chain polyphosphates (up to 700 phosphate units) that are responsible for improving epithelial barrier function *in vitro* and in mice [43–46]. Polyphosphate is endocytosed by the cell through caveolin-1 and integrin-β1 mechanisms and p38-MAPK-dependent gene expression and protein production of HSP27. Endocytosis is the key step for polyphosphate protective action [44, 45]. As a result, synthetic long-chain polyphosphates added to the diet may be serious candidates for mimicking the protective action of those probiotics *in vivo*.

*Collectively these data support the diet (including probiotics) as a major lever for stimulating gut defense systems and controlling inflammation and oxidative stress.*

### Gut IAP and iHSP defense systems and their nutritional modulation in the pig

The pig is a major source of meat worldwide and it is increasingly used as a biomedical model in various domains [13–16]. Most of the mechanisms of gut epithelial protection by IAP or iHSPs and their modulation by dietary components have been described, at least partly in the swine species too (e.g. for IAP: [47]).

#### Intestinal alkaline phosphatase

Pigs display three alkaline phosphatase gene copies in the intestine, thus being intermediate between domestic carnivorous (single copy) and ruminants (seven copies) [48]. IAP is strongly inhibited after early weaning in pigs and this is considered as a major factor in post-weaning disorders and enhanced piglet sensitivity to enteric infections [49]. The hormone glucagon-like peptide 2 (GLP-2), known for its intestine-trophic properties has been recently shown to stimulate duodenal and jejunal IAP in weaned pigs injected with exogenous (human) GLP-2 [50]. This was associated with the maturation of intestinal epithelial cells. Finally, piglets born to sows treated with antibiotics (amoxicillin) around parturition transiently displayed lower *Iap* gene expression and IAP enzyme activity than piglets born to untreated sows [51].

#### Inducible gut epithelial HSPs

Pigs display substantial and fairly similar iHSP concentrations in the small and large intestine [52–54], contrary to laboratory rodents that are virtually devoid of iHSPs in the small intestine (except in its distal part: the ileum) [34]. Growing pigs even displayed higher iHSP concentrations in the ileum than in the colon [55]. Intra-uterine growth retarded piglets were shown to display higher duodenal and jejunal HSP70, as hallmarks of fetal stress in utero [55]. iHSPs have been evidenced to be modulated by weaning along the GIT of piglets [52]. Small intestine iHSPs were not influenced in piglets born to sows given antibiotics (amoxicillin) around parturition but colonic HSP70 was transiently decreased [53]. Important links between iHSPs and the gut microbiota were demonstrated in pigs (fed chicory inulin, see below) [55]. These included: negative correlations between HSP27 and lumenal bacteria (*L. reuteri* and Enterobacteriacae), positive correlations between iHSPs and lactic acid-producing bacteria or *L. Johnsonii*. Ileal HSP27 and colonic HSP70 correlated negatively with the diversity of mucosa-associated bacteria and *Roseburia faecis* (a butyrate producer). Colonic HSP70 correlated negatively with *Prevotella brevis* but positively with the anti-inflammatory bacterium *Faecalibacterium prausnitzii* [56]. Although such individual correlations are difficult to interpret in terms of cause-and effect relationships, they suggest intimate interactions between iHSPs and the gut microbiota in pigs.

#### Dietary modulation of gut IAP and iHSPs in pigs

##### IAP

Few data are available on the effects of dietary factors on IAP in pigs [20, 21]. First, feed intake is an important IAP modulator in pig gut [53]. Regarding fat, Dudley et al. [57] reported higher IAP in pigs fed high fat diets with saturated (tallow), compared to unsaturated (corn oil) fat sources. Intestinal cell membranes reflected dietary fatty acid profiles, suggesting a link with IAP levels [43, 57]. Furthermore, wheat arabinoxylan alone or associated with cellulose was recently shown to increase ileal total AP activity [58]. This was interpreted as positive as it is essentially the IAP isoform that is present in the small intestine [20, 21]. The Authors also reported increased AP activity in the mid-colon in response to arabinoxylan supplementation [58]. This observation should be interpreted with caution because it was total AP (and not specifically IAP isoform) activity that was measured and this could reflect a sign of colonic inflammation, e.g. resulting from increased tissue infiltration by neutrophils [21]. Thus, effects of dietary components on GIT AP activity should be interpreted carefully according to GIT segment and efforts to differentiate between true IAP isoform and nonspecific AP activities using appropriate AP inhibitors [20] should be considered. Interestingly, IAP was shown to be higher in pigs selected for low, compared to high residual feed intake and this was associated with lower inflammation and circulating levels of LPS [59]. These data collectively suggest that IAP is influenced by the type/source of dietary fat and fiber and also reduces LPS intestinal translocation and inflammation in pigs. Also, intestinal IAP could be one key to residual feed intake and feed efficiency.

##### iHSPs

Feed intake modulates iHSPs along pig GIT [52]. Many feed ingredients, including amino acids and proteins, carbohydrates (including fiber) and fat are known to modulate gut function in pigs [18]. However, only some studies specifically investigated iHSPs.

L-glutamine as repeatedly been shown to improve growth performance and intestinal anatomy and function in weaned piglets [18], and these effects were partly mediated by intestinal epithelial HSP70 [60]. L-glutamine also improved intestinal maturation in intra-uterine growth retarded pig neonates through HSP70-mediated mechanisms [61]. Protective iHSP-mediated effects on the gut were also brought about with diets supplemented with L-arginine, α-ketoglutarate and N-carbamyl-glutamate [62, 63]. Besides, soybean proteins are considered as toxic for the gut of piglets [64]. The storage protein β-conglycinin was recently shown to inhibit gut HSP70 in pigs, probably contributing to the adverse effects of soybean proteins [65]. Conversely, a weaning diet supplemented with a melon pulp rich in the anti-oxidant enzyme superoxide dismutase decreased iHSP protein concentrations along the GIT of already weaned piglets, but this probably reflected reduced oxidative stress [66]. Finally, zinc oxide up-regulated *Hsp70* gene in porcine IPEC-J2 epithelial cell line but could not be shown to do so at high zinc level (2,200 ppm) *in vivo* [67–69]. Regarding dietary fiber, chicory pectin was recently shown to stimulate ileal and colonic HSP27 in growing pigs [56]. Interestingly, ileal iHSP27 was positively correlated with fiber intake and various correlations between iHSPs and the gut microbiota were set up for both the ileum and the colon (see above) [56]. Also, two probiotic strains (*L. johnsonii* strain P47-HY and *L. reuteri* strain P43-HUV) were demonstrated to stimulate iHSPs in IPEC-J2 porcine intestinal cell line *in vitro* [70]. By contrast, another probiotic (*Enterococcus faecium* strain NCIMB) did not do so in this porcine cell line, despite its stimulation on HSP70 in human Caco2 cells [71]. This highlights the host species-dependent specificity of probiotic effects on gut epithelial cells. Finally, we showed that the mycotoxin fumonisin-B1 slightly stimulated iHSP70 (but not iHSP27) in the jejunum, without effects on iHSPs in the colon of already weaned pigs [72].

## Conclusions

**Fig. 1:**
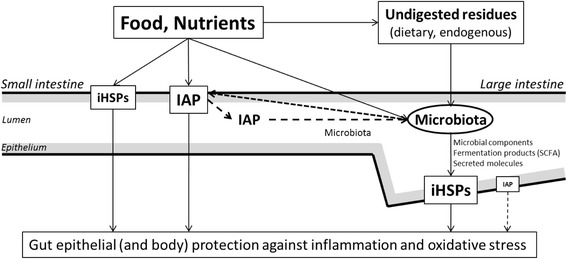
Various food components (nutrients, minerals, vitamins) modulate inducible heat shock proteins (iHSPs) and intestinal alkaline phosphatase (IAP) in the epithelium of the small intestine. It is mostly microbial compounds, fermentation products (short-chain fatty acids, SCFA) and other unknown secreted molecules of microbial origin that induce iHSP in the large intestine (nb: IAP expression and activity are very low there). Luminal IAP contributes to control the gut microbiota (present in low numbers) in the small intestine. Luminal IAP also partially escapes digestion in the small intestine and is active to shape the gut microbiota in the large intestine. iHSPs and IAP display potent anti-oxidant and anti-inflammatory properties that dynamically stimulate gut epithelial resistance to oxidative stress and inflammation. IAP is also anti-inflammatory systemically

The present review summarizes the published information on gut protection and defense systems, namely IAP and inducible HSPs, in rodent species and in pigs (Fig. 1). It also highlights the stimulation of these protection systems by a variety of dietary components that could, therefore be used to promote gut health. Importantly, many probiotic strains display protective properties that involve IAP and (or) iHSP stimulation. Data in pigs are more limited than in laboratory rodents but they also support roles for IAP and iHSPs in microbiota - host interactions and in controlling gut function and inflammation. Additional work is needed (especially in pigs) for setting up unequivocal cause-and effect relationships in the microbiota-host interaction for gut health and highlighting better the importance of dietary components for stimulating IAP- and (or) iHSP-dependent mechanisms of gut epithelial protection.

## Abbreviations

ATP: Adenosine triphosphate
CpG DNA: Cytosine-phosphate-guanidine deoxyribonucleic acid
ERGMT: Glutamyl-arginyl-glycyl-methionyl-threonine
ERK1/2: Extracellular signal-regulated kinase
fMLP: N-Formylmethionyl-leucyl-phenylalanine
GIT: Gastrointestinal tract
GPR: G-protein coupled receptor
HSF: Heat shock factor
HSP: Heat shock protein (iHSP, inducible HSP)
IAP: Intestinal alkaline phosphatase
LPS: Lipopolysaccharide
MAPK p38: p38 Mitogen-activated protein kinase
NF-κB: Nuclear factor-kappa B
OCTN-2: Organic cation transporter
PepT1: Peptide transporter 1
PUFA: Polyunsaturated fatty acid
SCFA: Short-chain fatty acid
TLR: Toll-like receptor
UDP: Uridine diphosphate
ZO: Zonula occludens
